# Morphine immunomodulation prolongs inflammatory and postoperative pain while the novel analgesic ZH853 accelerates recovery and protects against latent sensitization

**DOI:** 10.1186/s12974-019-1480-x

**Published:** 2019-05-21

**Authors:** Amy K. Feehan, James E. Zadina

**Affiliations:** 1 SE LA Veterans Health Care System, New Orleans, LA 70119 USA; 2 Dept. of Medicine, Neuroscience Lab #8516, 1430 Tulane Avenue, New Orleans, LA 70112 USA; 3 Brain Institute, Tulane University School of Medicine, New Orleans, LA 70112 USA

**Keywords:** Endomorphin, Latent sensitization, Chronic pain, Opioid, Morphine, Paw incision, Complete Freund’s adjuvant, Inflammation, pp38, Microglia

## Abstract

**Background:**

Numerous studies have identified the proinflammatory, pronociceptive effects of morphine which ultimately exacerbate pain. Our novel endomorphin analog ZH853 does not produce proinflammatory effects on its own and gives potent, long-lasting analgesia. This study investigates whether ZH853’s lack of interaction with the neuroimmune system reduces the risk of prolonged pain.

**Methods:**

Adult male Sprague-Dawley rats were subjected to one of two treatment paradigms. Either (1) chronic pain followed by chronic treatment with morphine, ZH853 or vehicle, or (2) chronic drug administered prior to pain induction. Complete Freund’s adjuvant (CFA) was injected or paw incision surgery was performed on the left hind plantar foot pad. Drugs were administered through Alzet osmotic minipumps at a rate of 1 μl/h for 5 days at appropriate doses based on prior experiments. Animals were tested for mechanical allodynia and thermal hyperalgesia using von Frey filaments and the Hargreaves apparatus, respectively. Additionally, several gait parameters were measured using the CatWalk XT. When all animals had recovered from pain, 1 mg/kg of naltrexone was administered to test for development of latent sensitization (LS). A second set of animals was used to investigate dorsal horn inflammation following CFA and drug treatment. ANOVAs were used to assess differences between drug treatment groups.

**Results:**

As expected, morphine increased and prolonged pain in all experiments compared to vehicle treatment. However, ZH853 treatment reduced the overall time spent in pain and the severity of pain scores compared to morphine. ZH853 not only reduced inflammation versus morphine treatment but also, in some instances, acted as an anti-inflammatory drug compared to vehicle treatment. Finally, ZH853 prevented the development of LS while vehicle- and morphine-treated animals showed robust relapse to pain.

**Conclusions:**

ZH853 has a favorable side effect profile versus morphine and provides superior analgesia in a number of pain states. We now know that chronic use of this compound *reduces* time spent in a chronic pain state, the opposite of common opioids like morphine, and reduces the risk of LS, making ZH853 an excellent candidate for clinical development in humans for inflammatory and postoperative pain.

## Background

In addition to the well-known negative side effects of currently used opioids, including abuse liability, respiratory depression, tolerance, and others, several recent studies have shown a lesser-known side effect; chronic exposure to morphine and other opioids can paradoxically exacerbate and prolong pain hypersensitivity. Known as the “two hit” hypothesis, it has been proposed that injury causes pro-inflammatory signaling in the central nervous system (CNS) which is exacerbated by morphine such that pain is ultimately more intense and longer-lasting [1, 2]. This exacerbation can occur with either order of stimulus (injury then drug or vice-versa) and can contribute to the transition from acute to chronic pain. The transition to chronic pain can also occur through another recently described mechanism known as “latent sensitization” (LS). LS is pathological pain following injury or inflammation that is “masked” during apparent recovery by endogenous opioid receptor function and “unmasked” by treatment with an opioid inverse agonist, such as naltrexone, or by stress [3–10]. In this study, we compare the effects of morphine and a novel opioid on both the paradoxical exacerbation of pain and on LS, with the goal of assessing the new analog for preventing the transition from acute to chronic pain.

We recently characterized a novel endomorphin [11] analog (ZH853) that shows antinociceptive effects equivalent to or greater than morphine, but with reduced abuse liability, respiratory depression, tolerance, hyperalgesia, impairment of motor coordination, and glial activation [11]. We also characterized the effectiveness of acute administration of the analog in alleviating several forms of chronic pain, including neuropathic, inflammatory, postoperative, and visceral pain [12]. This lead compound has been selected for development for clinical application and will be tested here for the effects of short term (3–5 days) chronic administration on the recovery from inflammatory and postoperative pain and the presence of LS.

In this study, drugs were chronically infused either before or after pain. Recovery from pain was measured to determine whether ZH853 causes detrimental effects on pain similar to those reported with morphine use in humans and animals [1, 2, 13].

The drug-then-pain paradigm has been studied by several groups in different pain states. Some chronic pain patients who use opioids therapeutically are known to experience worse pain following surgery than non-opioid users, and that pain cannot always be controlled satisfactorily [14, 15]. In rodent studies, Horvath et al. [16] showed that 7 or 14 days of chronic morphine infusion increased and prolonged both allodynia and thermal hyperalgesia, and the Watkins lab found that animals treated with morphine prior to chronic constriction injury or complete Freund’s adjuvant (CFA) had more intense and prolonged pain that correlated with a number of upregulated proinflammatory markers [2]. The reverse paradigm (pain then drug) is perhaps more clinically relevant but has been explored to a lesser extent. Recently, Grace et al. [1] found that morphine given after the induction of neuropathic pain prolonged hypersensitivity versus vehicle treatment, and this result was consistent with the effects of morphine treatment on postoperative pain [17]. Both studies found that morphine plus pain increased proinflammatory signaling in the spinal dorsal horn and that blocking inflammation pharmacologically blocks the effect of morphine on prolonged pain.

Comprehensively described by Taylor [8], Corder [4], and Marvizon [3, 6], LS and endogenous dependence are best thought of as two sides of the same coin; the phenomena are inextricably linked and cause a susceptibility to unmasking pain through either stress or chemical inactivation of Mu-opioid receptor (MOR) constitutive activity. The initial pain insult activates pain pathways (“accelerator”) and descending pathways induce constitutive activity at the MOR (MOR_CA_) to counteract pain (“brake”). Over a long period of recovery from the injury, the body becomes dependent on MOR_CA_, which causes increased sensitization of pain pathways (increasing the “brake” increases the “accelerator”). This interplay between increasing pain sensitization and increasing endogenous analgesia continues in a feed-forward cycle, establishing chronic, relapsing pain that is susceptible to stress [5, 9] or inverse agonists at the MOR [4, 6]. In fact, after an injury, descending pathways can be inhibited at the cervical spinal cord to induce a relapse to pain at the level of the lumbar spinal cord [3], indicating that descending modulation of opioid receptors and, to some extent, α2A adrenergic receptors is necessary to continually suppress pain [6]. LS has been demonstrated in humans [7], a subset of whom appear to be more prone to developing LS. As with inflammation, LS plays a role in the transition from acute to chronic pain.

In the current study, we examined the effects of morphine and ZH853 on two pain models (CFA and paw incision) in two long-term paradigms (drug then pain, pain then drug) using both traditional test methods (e.g., von Frey, Hargreaves, Randall-Selitto) as well as a functional assay (CatWalk XT), which examine different aspects of pain [18]. To assess inflammation, spinal cords were collected from a subgroup of animals and processed for immunohistochemistry when behavioral differences were most profound.

Finally, we tested the effects of morphine and ZH853 on LS induced by inflammatory (CFA) and postoperative (paw incision) pain. To do this, we used a standard 1 mg/kg injection of the MOR inverse agonist naltrexone (NTX) to shut off MOR constitutive activity, then measured mechanical allodynia, thermal hyperalgesia, and several CatWalk variables.

The results show that morphine exacerbated and prolonged chronic pain and had no effect on LS, while ZH853 accelerated time to recovery and blocked LS. These two findings in different paradigms indicate that ZH853 has a superior profile for reducing the transition from acute to chronic pain.

## Methods

### Animals

Male Sprague-Dawley rats (~ 59–67 days old and 250–300 g at the beginning of the experiments, Charles River, Wilmington, MA) were group housed in a 12-h light/dark cycle (6 am/6 pm) in a temperature- (68–72 °F) and humidity-controlled room with food and water provided ad libitum. All experiments were approved by the Tulane Institutional Animal Care and Use Committee and conducted according to the NIH Guide for the Care and Use of Laboratory Animals. All efforts were made to minimize animal suffering, and to reduce the number of animals used. No alternatives to in vivo techniques are available.

### Drugs

ZH853 was synthesized as described previously [11, 12] by Bachem (Torrance, CA). Morphine sulfate was supplied by NIDA. All drugs were dissolved in 20% polyethylene glycol (PEG) in sterile saline.

Drug injections for rats were given as described previously through intrathecal (i.t.) catheters [11, 12, 19]. Before drug dosing, rats with i.t. catheters underwent lidocaine testing to determine successful placement of the catheter. After a 10 μl injection of lidocaine followed by 12 μl of streptokinase (to maintain catheter patency), rats with properly placed catheters develop rapid but transient bilateral paralysis and recover in fewer than 5 min. Rats who did not recover or respond were immediately euthanized.

Osmotic minipumps (Alzet model 2001, Durect Corp, Cupertino, CA) filled with vehicle, morphine, or ZH853 and primed in 0.9% saline at 37 °C for 16 h, were implanted subcutaneously (s.c.), and connected to a PE-8 (0.008″ I.D.) i.t. catheter. In the pain-then-drug experiment, 80% of the *E*_max_ interpolated from dose response curves in [12] for CFA pain were given as a bolus dose (4.657 μg morphine, 0.06 μg ZH853) followed by infusion at a rate of 2.3285 μg/h of morphine and 0.02 μg/h of ZH853. Hourly dose was calculated as the bolus dose divided by the number of hours that the dose lasted. For ZH853, 0.06 μg lasted 3 h, so continual dosing was set at 0.02 μg/h. Then, 4.657 μg of morphine lasted 2 h, so 2.3285 μg/h was used as the hourly dose. Doses for postoperative pain were a bolus dose (1.932 μg morphine, 0.176 μg ZH853) followed by infusion at a rate of 1.932 μg/h of morphine and 0.07 μg/h of ZH853 for 3 days to comply with CDC guidelines for postoperative opioid use [20]. Hourly dose was calculated as the bolus dose divided by the number of hours that the dose lasted. For ZH853, 0.176 μg lasted 2.5 h, so continual dosing was set at 0.07 μg/h. Then, 1.932 μg of morphine lasted 1 h, so this was also used as the hourly dose. For the drug-then-pain experiment, the pumps delivered 8× the ED50/h (2 μg/h morphine [21], and 0.056 μg/h ZH853) for 5 days as determined previously to be equianalgesic in the tail flick assay [11]. Drug solutions were coded and the experimenter was blind to treatment.

### Adjuvant-induced inflammation

Hind paws were swabbed with a sterile 70% alcohol pad. As previously described [12, 22], CFA (100 μl, s.c., Sigma, St. Louis, MO) was injected into the left hindpaw using a 27-gauge needle. As an internal control, the rat’s right hind paw was injected with 100 μl of sterile saline. Testing started 24 h after injection. Before testing, swelling in the left paw was measured with a Plethysmometer Paw Volume Meter (IITC Life Science Inc., Woodland Hills, CA). Animals were monitored for signs of infection or axotomy at the injection site.

### Paw incision surgery

Hindpaws were swabbed with a sterile 70% alcohol pad. As previously described [12, 23], a 1-cm longitudinal incision was made with a No. 11 blade through the skin and fascia on the plantar aspect of the left hindpaw beginning 0.5 cm from the end of the heel. The flexor muscle was elevated with forceps and incised longitudinally several times. The skin was closed with two 5–0 surgical sutures (Ethicon, Somerville, NJ). Behavioral testing started 1 h after wound closure.

### Pain assessments

All animals were monitored for signs of axotomy, infection, or porphyrin staining which would indicate extreme stress and exclude them from behavioral testing. All tests were started in the morning, beginning with the least noxious (CatWalk and von Frey) and followed by Hargreaves and Randall-Selitto testing with at least 20 min of acclimation between tests. Animals were acclimated for at least 30 min prior to von Frey testing at the beginning of the test period. Baseline measurements were conducted after the i.t. catheter was implanted. Animals were randomized into drug groups by an experimenter blinded to drug treatment such that average baselines for each group were as similar as possible across all baseline tests. All animals were used only once to prevent drug or testing experience from confounding the study and were drug- and test-naïve when the study started.

### Mechanical allodynia

Mechanical allodynia was assessed using nylon von Frey filaments (Stoelting, Wood Dale, IL) according to the “up-down” algorithm described by Chaplan [24]. The apparatus and procedure are described elsewhere [12]. Briefly, rats were placed on wire mesh platforms in plexiglass boxes for 30 min of acclimation. Fibers of increasing stiffness were applied dorsally just lateral to the paw incision, pressed upward to cause a slight bend in the fiber and left in place for 8 s [25]. The experimenter was careful to avoid the toes and hairline of the paw. Withdrawal of the hind paw from the fiber was scored as a response. When no response was obtained, the next stiffest fiber in the series was applied to the same paw; if a response was obtained, a less stiff fiber was applied. Testing proceeded in this manner until four fibers had been applied after the first one causing a withdrawal response, allowing the estimation of the mechanical withdrawal threshold. Sensory thresholds were estimated as described previously [24]. This assay can detect mechanical thresholds as low as 0.02 g.

### Thermal hyperalgesia

Withdrawal latency to heat was evaluated using the IITC Plantar Analgesia Meter (IITC Life Science, Inc.) to assess thermal hyperalgesia [12, 26]. In this procedure, a radiant heat source was directed at the hind paws (intensity 70, cutoff 15 s) and latency to withdraw was recorded. A high intensity projector bulb (Osram 58–8007 8 V, 50 W) positioned 40 mm under a glass floor was projected through a 5 × 10 aperture in the top of a movable case. Once the rat withdrew the hindpaw, the heat source was turned off and latency to withdraw was recorded three times for each paw and averaged.

### CatWalk XT

To measure functional impairment and recovery from inflammation, we used the CatWalk XT version 10.6 gait analysis system (Noldus Information Technology, Wagening, Netherlands), which has been described in detail elsewhere [27–31]. Briefly, paw prints are captured by a high-speed video camera positioned underneath a long narrow plexiglass-bottomed chamber. The rat is put in one end of the chamber and crosses it to reach a dark chamber that leads to the animal’s home cage. Contact with the plexiglass causes a distortion of light that the system then interprets to track and analyze paw prints. Three compliant runs were collected at each time point with a maximum run time of 5 s (average = 1.52 ± 0.03 s) and run variation of less than 60% (average = 28 ± 0.79%). These parameters were sufficient to produce smooth, consistent runs across the experiment. The CatWalk XT also has an automatic classification system to define all four paws. A researcher not involved in data analysis went through each compliant run to filter out false signals or errant classifications and exclude partial paw prints at the beginning and ending of each run. We investigated the following parameters:

- Swing time is the duration of no contact with the glass plate per step cycle and has been shown to increase in pain conditions.
- Swing speed is the rate in meters/second that a paw is not in contact with the glass plate.
- Stand time is the duration of ground contact for a single paw. This variable will decrease with pain and is the counterpart to the swing phase.
- Duty cycle expresses the stand as a percentage of a step cycle. Duty cycle = stand/(stand + swing) × 100%.
- Single stance measures the amount of time that the left hind paw is contacting the glass in the absence of the right hind also touching the glass. This will decrease in pain conditions.
- Paw print length which is the length from the center toe to the farthest central point of the print. Print length decreases in pain conditions.
- Maximum contact intensity of a paw which is an indirect measure of how much weight the animal bears on that paw. Weight bearing, and therefore contact intensity, decrease in pain states.

### Naltrexone-precipitated unmasking of pain

Regardless of the order of intervention or pain type, all animals recovered to baseline, but dysregulation of the endogenous opioid system by morphine, a pain state, or both has been documented to “mask” the actual pain state [5–7, 9, 32, 33]. By using naltrexone 1 mg/kg s.c., we probed whether drug, pain, or drug plus pain groups showed an unmasking of latent sensitization. Animals were given naltrexone and testing occurred 30–60 min after injection, at a time when naltrexone activity is stable. All testing techniques described in Marvizon 2015 [10] were followed as closely as possible except that animals were not tested over a time course.

### Immunohistochemistry

When behavioral scores were most different, 25 days after CFA injection in the first paradigm (pain then drug) and 21 days after CFA injection in paradigm 2 (drug then pain), a subset of animals were deeply anesthetized with ketamine/xylazine (85/10 mg/kg) and transcardially perfused with 0.1 M phosphate-buffered saline (PBS) followed by 4% paraformaldehyde. Spinal cords were collected and post-fixed overnight at 4 °C, cryoprotected in 30% sucrose/0.1 M PBS for 2 days, and sectioned in 50 μm slices using a cryostat. Spinal cord sections from L4-L5 were collected and every 6th section was developed with a different antibody stain or combination of two stains. After two washes in PBS and blocking with 5% normal horse serum/0.3% Triton X-100, sections were incubated in the following primary antibodies: calcitonin gene-related peptide (CGRP) (rabbit, T-4032, Peninsula Labs, San Carlos, CA), glial fibrillary acidic protein monoclonal (GFAP) (mouse, Astro6 MA5-12023, ThermoFisher, Carlsbad, CA), Anti-CD11b/c (OX42) (rabbit, CBL1512-100UG, Millipore Sigma, St. Louis, MO), purinergic receptor 7 (P2X7R) (rabbit, #APR-008, Alomone Labs, Jerusalem, Israel), phosphorylated-p38 MAP kinase (pp38) (rabbit, #4511, Cell Signaling Technology, Danvers, MA), or interleukin-1beta (IL-1β) (rabbit, ab9787, Abcam, Cambridge, MA) and were incubated for 24 h at 4 °C on a slow rocker. All tissue was washed twice, re-blocked with serum, and incubated in donkey anti-mouse secondary antibody conjugated to Alexa488 (A21202, ThermoFisher) or donkey anti-rabbit conjugated to Alexa594 (A21207, ThermoFisher) for 2 h at room temperature, washed, and slide mounted with VECTASHIELD Antifade mounting medium with DAPI (H-1200, Vector Laboratories, Burlingame, CA). For all negative controls, the same procedure was performed but the primary antibody was omitted to check for non-specific staining.

### Imaging and analysis

All images were captured with a Nikon Ni-E microscope and Hammamatsu camera. ImageJ software was used to assess integrated density as previously described [11]. Briefly, a blinded observer set thresholds on images with the default ImageJ algorithm and calculated the integrated density (area times mean gray value) in a specified region of interest. The gray value includes both the intensity and number of pixels above the set threshold in the region of interest. This controls for background staining variability. When counting stain-positive cells, a blinded observer used ImageJ to count cells from five randomly selected viewing fields within the region of interest, and a second blinded observer confirmed these counts. At least 4–6 rats per group, per endpoint were used for immunohistochemistry (IHC) experiments, and 4–6 slices per rat (right and left dorsal horns) were quantified for each experiment. There were no differences between right and left dorsal horns, so data was collapsed into one column.

### Statistical analysis

Data sets were analyzed with Prism (GraphPad Software, La Jolla, CA) and are expressed as mean ± standard error of the mean (SEM). Animal numbers of 4–7 per group were used based on similar experiments in our lab and others’ for appropriate statistical analysis. For all time course data, two-way repeated measures analysis of variance (ANOVA)s followed by Newman-Keuls post-hoc testing were used to determine differences in drug group over time. In the recovery analysis, the same analysis was used to compare each point to the baseline. The time point when test scores are not significantly different from baseline was considered “recovery.” Areas under the curve (AUC) were calculated for each time course for each animal. Differences between group means were determined by one-way ANOVA with Newman-Keuls post-hoc test.

## Results

**Fig. 1:**
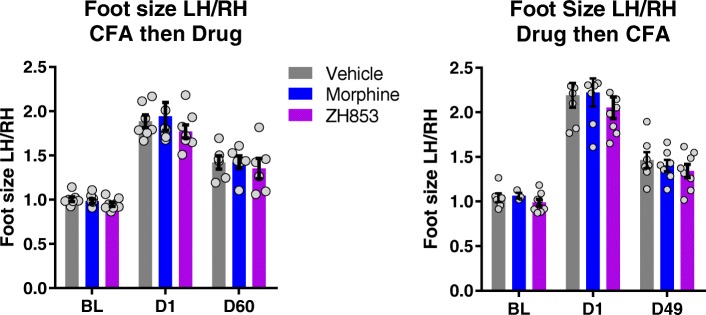
Plethysmometer data. Left hind (LH) paw over right hind (RH) paw values for foot size. Significant differences (*p* < 0.01) were observed among the three time points, but not among the drugs or the interaction, indicating a consistent injury across groups

Paw volumes for the CFA experiment, reflecting inflammation-induced edema, were determined by plethysmometer, and left hind (LH)/right hind (RH) values were calculated for vehicle, morphine, and ZH853 groups prior to CFA and after injection. There were no significant differences between groups (Fig. 1). Despite the consistency of injury, morphine caused increased sensitivity in all tests compared to vehicle treatment while ZH853 did not.

### CFA then drug

#### Mechanical allodynia and naltrexone unmasking

**Fig. 2:**
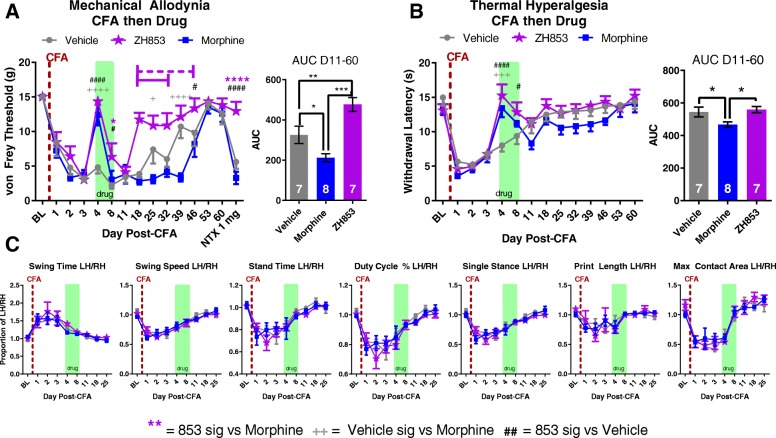
CFA then drug. Drug dosing is indicated with green boxes (5 days of drug (D3–8), 2 days wash out (D9–10)) and CFA injection is indicated by the red dashed line. “Days” indicate days after CFA. **a** Mechanical allodynia was increased by morphine treatment and decreased by ZH853 treatment relative to vehicle treatment. **b** Thermal hyperalgesia was increased by morphine treatment. ZH853 and morphine reversed hypersensitivity in both von Frey (green box in A) and Hargreaves (green box in **b**) tests 24 h after implantation of the minipumps, and both drugs were less effective by day 5 of infusion (day 8). **c** In the CatWalk test, all variables are a proportion of left hind paw (LH) values over right hind paw (RH). Drug treatment did not alter the functional impairments caused by CFA and all animals recovered from gait disturbances by the end of drug treatment. n indicated in bar graphs. Dashed lines indicate difference versus morphine while solid lines indicate difference versus vehicle. Two-way, repeated measures ANOVAs were used in all cases. Error bars indicate SEM. + = veh vs morphine, # = veh vs ZH853, * = morphine vs 853. *p < 0.05; **p < 0.01; ***p < 0.001; ****p < 0.0001

After treating CFA-induced mechanical allodynia with vehicle or equi-antinociceptive doses of morphine or ZH853, animals treated with morphine experienced greater allodynia than vehicle-treated animals at 25, 39, and 46 days after CFA injection (Fig. 2a). ZH853-treated animals, however, recovered to baseline by day 18 and experienced less allodynia than vehicle from day 18 to 32 and less than morphine-treated animals from day 18 to 46 (interaction *F*_28, 266_ = 4.681, *p* < 0.0001; time *F*_14, 266_ = 35.29, *p* < 0.0001; drug *F*_2, 19_ = 14.09; *p* = 0.0002). The AUC between day 11 and 60 shows that morphine-treated animals had significantly more allodynia than vehicle- and ZH853-treated groups, and that vehicle-treated animals had more overall pain than ZH853 animals (*F*_2, 19_ = 15.61, *p* < 0.0001). At day 53 and 60, all groups had returned to baseline allodynia, and a naltrexone injection was used to probe whether LS had developed. In both vehicle (*p* < 0.0001) and morphine (*p* < 0.0001) groups, significant allodynia developed after 30 min, but ZH853-treated animals showed no change in allodynia. Additionally, on the last day of drug dosing (D8, green box), ZH853 still produced some anti-allodynia which was not the case for morphine, indicating reduced tolerance caused by ZH853.

#### Thermal hyperalgesia

After the development of thermal hyperalgesia, drug dosing with either morphine or ZH853 reversed pain on the first day of drug dosing (D4, green box) (Fig. 2b). This effect diminished by day 8, but to a lesser extent in the ZH853 group. Although no specific time point was significantly different between groups during recovery, there were significant differences in the time by drug interaction (interaction *F*_26, 247_ = 2.223, *p* = 0.0009; time *F*_13, 247_ = 38.99, *p* < 0.0001; drug *F*_2, 19_ = 2.624, *p* = 0.0986). Additionally, the AUC indicates that morphine-treated animals experienced more sensitivity overall (*F*_2, 19_ = 4.763; *p* = 0.0211) (Fig. 2b).

#### Functional impairment

In the analysis of seven CatWalk variables, changes in gait function were apparent following CFA injection but, because drug dosing occurred when most of these variables had nearly returned to baseline, there were no differences between groups due to drug treatment (swing time interaction *F*_16, 152_ = 0.3986, *p* = 0.9813; time *F*_8, 152_ = 19.04, *p* < 0.0001; drug *F*_2, 19_ = 0.7010, *p* = 0.5085) (Fig. 2c). Additionally, LH/RH values for each variable were stable around 1 from day 18 onward and did not change following naltrexone injections (data not shown).

#### Prolonged pain

By comparing the means at each time point with the group baseline using a two-way ANOVA, we determined the average length of time in pain by drug treatment groups from day 11 to the end of the study. Significance is listed in Table 1. Morphine significantly prolonged allodynia and thermal hyperalgesia versus both vehicle and ZH853. ZH853, on the other hand, shortened allodynia and thermal hyperalgesia versus both morphine and vehicle. Drug administration did not affect CatWalk variables, likely because animals had nearly returned to baseline by the onset of drug treatment.

**Table 1:**
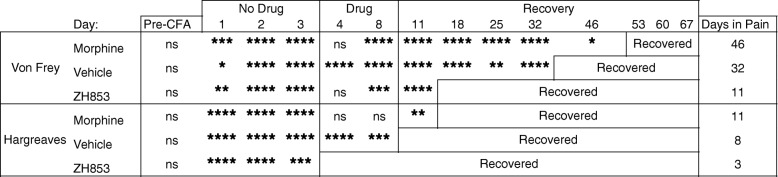
Time to recovery analysis: CFA then drug

Comparison of each time point to the group baseline to determine when that group recovered (difference from baseline ns). Total time of significant hypersensitivity (days in pain) is noted in the right column. Morphine prolonged recovery compared to vehicle and ZH853 treatment. ZH853 expedited recovery versus vehicle and morphine treatment. “Day” indicates the number of days since CFA injection. Two-way ANOVAs for von Frey and Hargreaves were conducted separately. *p < 0.05, **p < 0.01, ***p < 0.001, ****p < 0.0001

### Drug then CFA

#### Mechanical allodynia and naltrexone unmasking

**Fig. 3:**
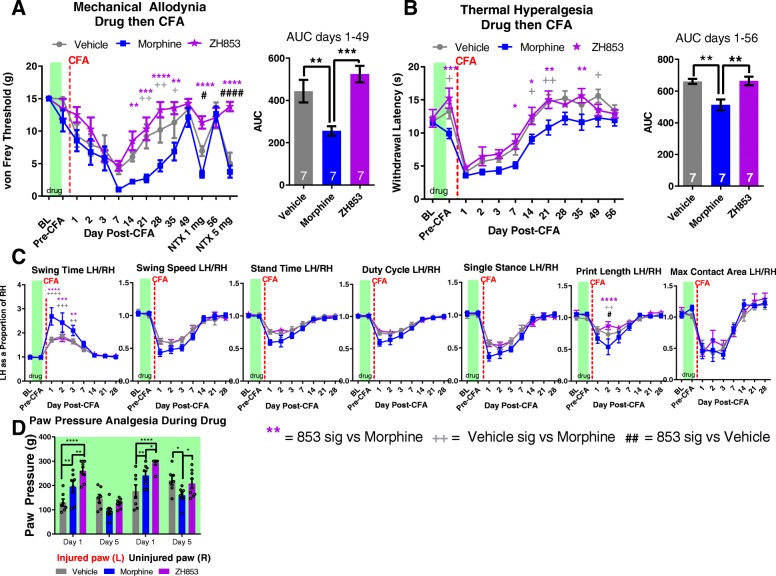
Drug then CFA. Drug dosing is indicated with green boxes (5 days of drug, 2 days wash out) and CFA injection is indicated by red line. “Days” indicate days after CFA. **a** Mechanical allodynia and **b** thermal hyperalgesia were prolonged by prior exposure to morphine but not ZH853, despite the fact that ZH853 produced greater antinociception in the paw pressure test 24 h after implantation of the minipumps (**d**). In the CatWalk test (**c**), morphine-treated animals showed exaggerated functional impairment (guarding the left paw) within the first few days after CFA injection versus vehicle- and ZH853-treated animals. *n* = 7 for all groups. Error bars indicate SEM. + = veh vs morphine, # = veh vs ZH853, * = morphine vs 853.*p < 0.05; **p < 0.01; ***p < 0.001; ****p < 0.0001

After chronic drug dosing, the subsequent CFA injection induced mechanical allodynia over the first 7 days. Animals treated with morphine experienced greater allodynia than vehicle-treated animals at 21, 28, and 35 days after CFA injection (Fig. 3a). ZH853-treated animals, however, experienced less pain than the morphine group from day 14 to 35 (interaction *F*_26, 234_ = 2.399, *p* = 0.0003; time *F*_13, 234_ = 21.10, *p* < 0.0001; drug *F*_2, 18_ = 18.87, *p* < 0.0001). The AUC between day 1 and 49 shows that morphine animals had significantly greater allodynia than vehicle and ZH853 groups, and that vehicle animals had similar pain to ZH853 animals (*F*_2, 18_ = 12.22, *p* = 0.0004). At day 49, all groups had returned to baseline allodynia, and a naltrexone injection (1 mg/kg) was used to probe whether latent sensitization had developed. In both vehicle- and morphine-treated animals, significant allodynia developed after 30 min, but ZH853-treated animals showed no significant change in allodynia after either 1 or 5 mg/kg of naltrexone. After the 1 mg/kg injection of naltrexone, allodynia was slightly increased in morphine versus vehicle-treated animals, but at 5 mg/kg a floor effect made them equally sensitive (see graph for statistics).

#### Thermal hyperalgesia

After pre-treatment with drug, thermal hyperalgesia developed. Animals treated with morphine experienced greater hyperalgesia than vehicle-treated animals at 14, 21, and 49 days after CFA injection (Fig. 3b). ZH853-treated animals, however, experienced less pain than the morphine group from day 7–21 and on day 35 (interaction *F*_22, 198_ = 1.027 *p* = 0.4331; time *F*_11, 198_ = 53.68, *p* < 0.0001; drug *F*_2, 18_ = 10.75, *p* = 0.0008). The AUC between day 1 and 56 shows that morphine animals had significantly more pain than vehicle and ZH853 groups, and that vehicle animals had similar pain to ZH853 animals (*F*_2, 18_ = 10.42, *p* = 0.0010). Notably, morphine-treated animals were significantly more sensitive than vehicle and ZH853 groups when tested just prior to CFA injection 2 days after cessation of drug dosing, which is not unusual given morphine’s proinflammatory and pain sensitizing effects after chronic dosing (see figure for statistics).

#### Functional impairment

In the analysis of seven CatWalk variables, changes in gait were apparent following CFA injection, and made worse by pre-treatment with morphine for the first few days (Fig. 3c). Morphine animals had greatly increased swing time, the amount of time that the paw is held in the air, for the first 3 days after CFA injection (interaction *F*_16, 144_ = 2.889, *p* < 0.0004; time *F*_8, 144_ = 40.6, *p* < 0.0001; drug *F*_2, 18_ = 4.061; *p* = 0.0350). Print length was shortened in the first few days after injury; even more so in morphine-treated animals (interaction *F*_16, 144_ = 2.426, *p* = 0.0029; time *F*_8, 144_ = 33.75; *p* < 0.0001; drug *F*_2, 18_ = 1.693, *p* = 0.2119). All animals had a decrease in swing speed after injury, the rate in meters/second that a paw is not in contact with the glass plate (interaction *F*_16, 144_ = 1.590, *p* = 0.0782; time *F*_8, 144_ = 81.14; *p* < 0.0001; drug *F*_2, 18_ = 1.099, *p* = 0.3545). Stand time, or duration of ground contact, was also decreased by injury (interaction *F*_16, 144_ = 1.253, *p* = 0.2354; time *F*_8, 144_ = 46.16; *p* < 0.0001; drug *F*_2, 18_ = 2.817, *p* = 0.0863). Duty cycle (interaction *F*_16, 144_ = 1.601, *p* = 0.0753; time *F*_8, 144_ = 64.26; *p* < 0.0001; drug *F*_2, 18_ = 2.277, *p* = 0.1314), single stance (interaction *F*_16, 144_ = 1.258, *p* = 0.2324; time *F*_8, 144_ = 76.66; *p* < 0.0001; drug *F*_2, 18_ = 1.383, *p* = 0.2762), and maximum contact area (interaction *F*_16, 152_ = 0.6678, *p* = 0.8220; time *F*_8, 152_ = 47.76; *p* < 0.0001; drug *F*_2, 19_ = 0.2218, *p* = 0.8031) were also decreased within the first few days. (LH/ RH values for each variable were back to baseline from day 14 onward and did not change following naltrexone injections of either 1 mg/kg or 5 mg/kg (data not shown). Data are summarized in Table 2.

**Table 2:**
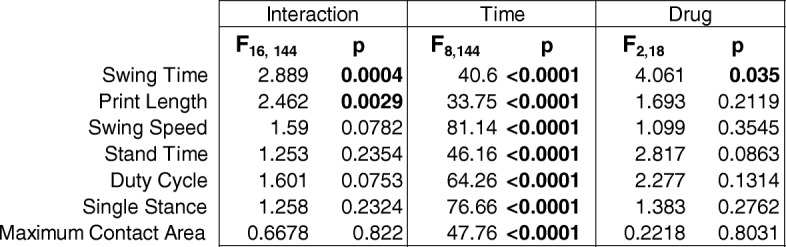
Drug then CFA CatWalk Statistics

*F* and *p* values for each CatWalk variable. Bolded values indicate significance as determined by two-way, repeated measures ANOVAs

#### Duration of allodynia and hyperalgesia

Means were compared between each time point and the group baseline using a two-way ANOVA to determine the average length of time in pain by drug treatment groups from day 1 to the end of the study. Significance is listed in Table 3. Morphine significantly prolonged allodynia versus both vehicle and ZH853. ZH853 shortened allodynia and thermal hyperalgesia versus both morphine and vehicle.

**Table 3:**
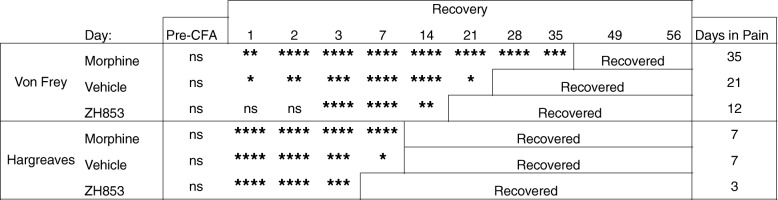
Time to recovery analysis: drug then CFA

Comparison of each time point to the group baseline to determine when that group recovered. Total time of significant hypersensitivity (days in pain) is noted in the right column. Morphine prolonged recovery (von Frey) or had no impact on time to recovery (Hargreaves) compared to vehicle treatment. However, ZH853 expedited recovery versus vehicle and morphine treatment. “Day” indicates the number of days since CFA injection. Drug was given for 5 days and stopped for 2 days prior to CFA. Two-way ANOVAs for von Frey and Hargreaves separately. *p < 0.05, **p < 0.01, ***p < 0.001, ****p < 0.0001

### Paw incision then drug

#### Mechanical allodynia

**Fig. 4:**
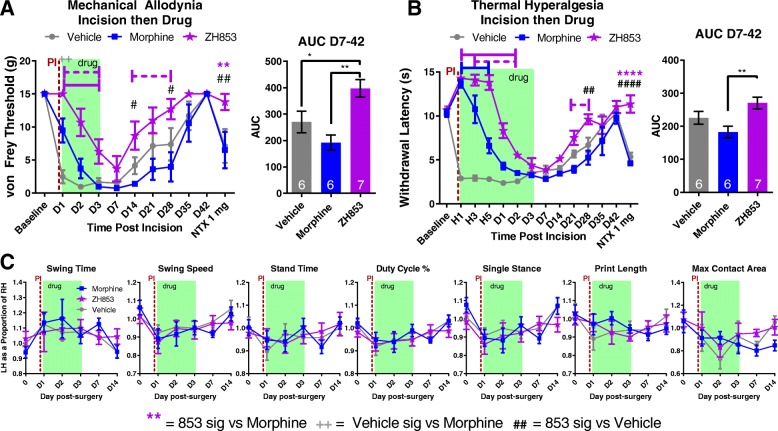
Paw incision (PI) then drug. Immediately after closing the incision, a bolus injection of drug was given and pumps were connected for 3 days. **a** Mechanical allodynia was greatly reduced by both morphine and ZH853 on the first day of drug but by day 2 and 3 only ZH853 gave analgesia versus vehicle treatment. **b** ZH853 prevented thermal hyperalgesia for longer than morphine, but ultimately animals tolerated to both drugs by D3. However, ZH853-treated animals recovered more quickly than vehicle or morphine groups. Upon treatment with naltrexone, relapse to thermal hyperalgesia was apparent in vehicle- and morphine-treated animals but not ZH853-treated animals. **c** CatWalk data was highly variable and there were no differences between drug treatment groups. n indicated in bar graphs. Dashed lines indicate difference versus morphine while solid lines indicate difference versus vehicle. Error bars indicate SEM. + = veh vs morphine, # = veh vs ZH853, * = morphine vs 853. *p < 0.05; **p < 0.01; ***p < 0.001; ****p < 0.0001

After treating paw incision-induced mechanical allodynia with vehicle or equi-antinociceptive doses of morphine or ZH853, animals treated with morphine experienced greater allodynia overall than vehicle-treated animals (Fig. 4a). ZH853-treated animals, however, experienced less pain than vehicle on days 14 and 28, and less pain than morphine-treated animals from day 14 to 28 (interaction *F*_20, 160_ = 2.976, *p* < 0.0001; time *F*_10, 160_ = 32.82, *p* < 0.0001; drug *F*_2, 16_ = 12.64, *p* = 0.0005). The AUC between day 7 and 42 shows that morphine-treated animals had significantly more pain than vehicle- and ZH853-treated groups, and that vehicle-treated animals had more overall pain than ZH853-treated animals (*F*_2, 16_ = 9.293, *p* = 0.0021). At day 42, all groups had returned to baseline allodynia, and a naltrexone injection was used to probe whether latent sensitization had developed. In both vehicle- (*p* < 0.001) and morphine- (*p* < 0.001) treated animals, significant allodynia developed after 30 min, but ZH853-treated animals showed no change in allodynia. Additionally, on the last day of drug dosing (D3, green box), ZH853 still produced some anti-allodynia versus vehicle which was not the case for morphine.

#### Thermal hyperalgesia

Morphine and ZH853 were antinociceptive in the first hour of drug dosing (Fig. 4b: H1, green box), but morphine antinociception was greatly reduced at 3 and 5 h. By D1, morphine-treated animals had pain similar to vehicle-treated animals, but ZH853 was still providing some pain relief. By D3, all groups were similarly hyperalgesic. On days 21–28, morphine-treated animals were in more pain than ZH853-treated animals, and on day 28, vehicle-treated animals were also in more pain than ZH853-treated animals (interaction *F*_26, 208_ = 14.56, *p* < 0.0001; time *F*_13, 208_ = 47.27, *p* < 0.0001; drug *F*_2, 16_ = 60.74, *p* < 0.0001). After all groups were recovered at D42, naltrexone was administered and thermal hyperalgesia developed in vehicle- and morphine-treated animals but not ZH853-treated animals (*p* = 0.0001). Additionally, the AUC indicates differences in sensitivity over the recovery time course (*F*_2, 16_ = 5.970, *p* = 0.0116) with morphine-treated animals experiencing the most sensitivity followed by vehicle- then ZH853-treated animals.

#### Functional impairment

In the analysis of seven CatWalk variables, changes in gait function were apparent following CFA injection, but there were no differences between drug treatment groups (swing time, interaction *F*_10, 75_ = 0.9907, *p* = 0.4591; time *F*_5, 75_ = 4.082, *p* = 0.0025; drug *F*_2, 15_ = 2.062, *p* = 0.1617) (Fig. 4c). It should be noted that the CatWalk only captures subtle changes in gait following paw incision surgery unlike the dramatic changes seen with CFA. The small dynamic range in these gait changes may make it more difficult to see differences in drug treatment groups.

#### Prolonged allodynia and hyperalgesia

By comparing the means at each time point with the group baseline using a two-way ANOVA, we determined the average length of time in pain by drug treatment groups from day 1 to the end of the study. Significance at each time point is listed in Table 4. Morphine significantly prolonged thermal hyperalgesia versus both vehicle and ZH853. ZH853, on the other hand, shortened allodynia and thermal hyperalgesia versus both morphine and vehicle. Drug administration did not affect CatWalk variables, likely because they had nearly returned to baseline by the onset of drug treatment.

**Table 4:**
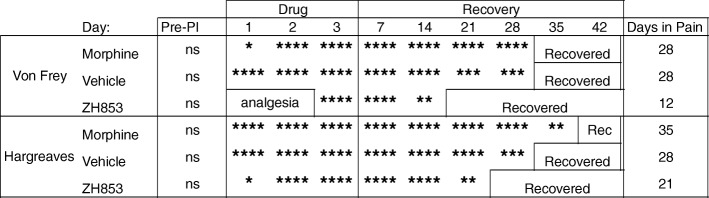
Time to recovery analysis: paw incision (PI) then drug

Comparison to baseline. Total time of significant hypersensitivity (days in pain) is noted in the right column. ZH853 reduced the time to recovery for allodynia and thermal hyperalgesia versus vehicle and morphine treatment. Morphine prolonged thermal hyperalgesia. “Day” indicates the number of days since paw incision. Two-way ANOVAs for von Frey and Hargreaves were conducted separately. *p < 0.05, **p < 0.01, ***p < 0.001, ****p < 0.0001

### Drug then paw incision

#### Mechanical allodynia

**Fig. 5:**
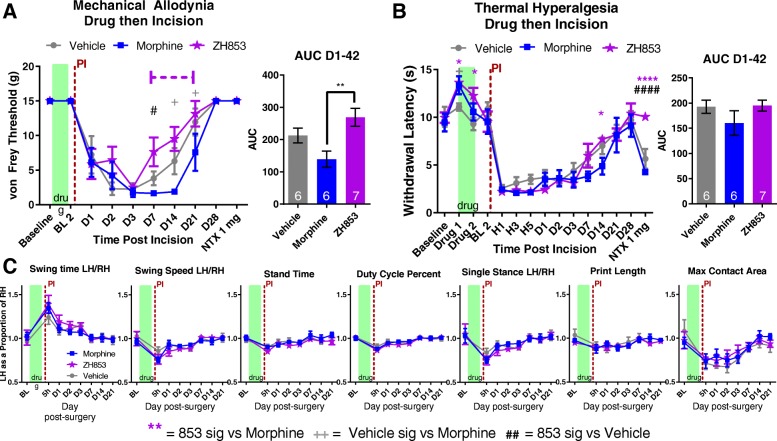
Drug then paw incision (PI). “Days” indicate days after paw incision. Drug was given for 5 days and stopped for 2 days prior to incision. **a** Mechanical allodynia developed in all groups following paw incision, but ZH853-treated animals recovered faster than vehicle- or morphine-treated animals. **b** Thermal hyperalgesia was severe and long-lasting in all drug treatment groups, but ZH853 prevented LS as indicated by lack of unmasking by naltrexone. **c** CatWalk variables changed in response to paw incision but not by drug group. n indicated in bar graphs. Dashed lines indicate difference versus morphine. Error bars indicate SEM. + = veh vs morphine, # = veh vs ZH853, * = morphine vs 853. *p < 0.05; **p < 0.01; ***p < 0.001; ****p < 0.0001

After exposure to tolerance-inducing doses of morphine or ZH853, animals treated with morphine experienced greater allodynia than vehicle-treated animals at 14 and 21 days after paw incision (Fig. 5a). ZH853-treated animals, however, showed improved pain sensitivity versus vehicle- and morphine-treated animals as early as D7, and experienced less pain than morphine-treated animals from D7 to 21 (interaction *F*_18, 144_ = 1.957, *p* = 0.0158; time *F*_9, 144_ = 57.06, *p* < 0.0001; drug *F*_2, 16_ = 2.540, *p* = 0.1102). The AUC between D1 and 42 shows that morphine animals had significantly greater allodynia than ZH853-treated animals (*F*_2, 16_ = 6.509, *p* = 0.0085). At day 28, all groups had returned to baseline, and a naltrexone injection was used to probe whether latent sensitization had developed. Unexpectedly, no allodynia was observed in any group at this time point.

#### Thermal hyperalgesia

After drug treatment, paw incision induced a strong thermal hypersensitivity in all animals. There were significant differences by the interaction of time and drug group (interaction *F*_28, 224_ = 2.697, *p* < 0.0001; time *F*_14, 224_ = 79.95, *p* < 0.0001; drug *F*_2, 16_ = 1.433, *p* = 0.2676) and a naltrexone challenge caused thermal hyperalgesia in vehicle- and morphine-treated groups but not ZH853-treated groups (*p* < 0.0001). The AUC indicates a lack of overall differences in sensitivity (*F*_2, 16_ = 1.324, *p* = 0.2937) (Fig. 5b).

#### Functional impairment

In the analysis of seven CatWalk variables, changes in gait function were apparent following paw incision, but there were no differences between groups due to drug treatment (swing time: interaction *F*_14, 112_ = 0.7318, *p* = 0.7385; time *F*_7, 112_ = 19.82, *p* < 0.0001; drug *F*_2, 16_ = 0.5549, *p* = 0.5848) (Fig. 5c).

#### Prolonged allodynia and hyperalgesia

By comparing the means at each time point with the group baseline using a two-way ANOVA, we determined the average length of time in pain by drug treatment groups from D1 to the end of the study. Significance at each time point is listed in Table 5. Morphine significantly prolonged allodynia versus both vehicle and ZH853. ZH853, on the other hand, shortened allodynia vs morphine and thermal hyperalgesia versus both morphine and vehicle.

**Table 5:**
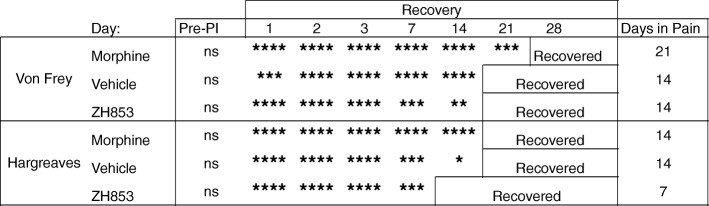
Time to recovery analysis: drug then paw incision (PI)

Comparison to baseline. Total time of significant hypersensitivity (days in pain) is noted in the right column. Pre-treatment with ZH853 reduced time to recovery for thermal hyperalgesia versus vehicle, and morphine prolonged mechanical allodynia versus vehicle and ZH853. “Day” indicates the number of days since paw incision (PI). Drug was given for 5 days and stopped for 2 days prior to PI. Two-way ANOVAs for von Frey and Hargreaves separately. *p < 0.05, **p < 0.01, ***p < 0.001, ****p < 0.0001

### Inflammation

In order to determine whether morphine-exacerbated CFA pain is associated with potentiated pro-inflammatory signaling in the spinal dorsal horn, spinal cords were collected from animals perfused at 25 days (CFA then drug) and 21 days (drug then CFA) for IHC. These time points were chosen because of the profound differences in allodynia between drug treatment groups. Several measures of inflammation were assessed at the level of L4–L6 of the spinal cord. Overall, inflammatory markers were upregulated consistently among morphine-treated animals. In almost every instance, ZH853-treated animals had reduced inflammation when compared with morphine-treated animals, and vehicle-treated animals had variable inflammation in relation to morphine or ZH853.

**Fig. 6:**
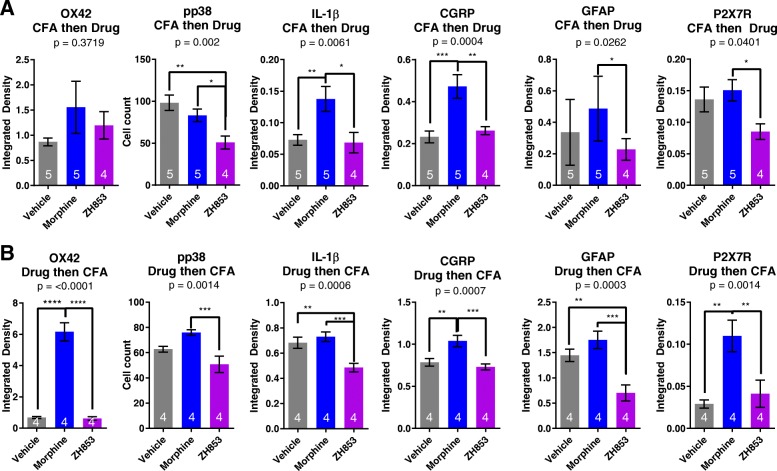
Immunohistochemistry following CFA and drug. Tissue was taken 25 days after CFA in the CFA-then-drug paradigm and 21 days after CFA in the drug-then-CFA paradigm. **a** CFA then drug. Staining at this time point did not show higher microglial activation in any group, but pp38 was decreased by ZH853, and IL-1β and CGRP were increased by morphine relative to vehicle and ZH853. Astrocyte activation and P2X7Rs were increased in morphine-treated animals compared to ZH853-treated animals. **b** Drug then CFA. Microglial activation was greater in animals pretreated with morphine- versus vehicle- and ZH853-treated animals. pp38 was increased by morphine and decreased by ZH853, producing a significant difference between drugs. IL-1β was significantly decreased by ZH853 relative to morphine and vehicle. CGRPR staining was increased by morphine relative to vehicle- or ZH853-treated animals. Astrocyte activation was decreased by ZH853 relative to vehicle and morphine, and P2X7R was increased by morphine relative to vehicle and ZH853. n indicated in bar graphs. Error bars indicate SEM. One-way ANOVAs were performed for each marker. *p < 0.05; **p < 0.01; ***p < 0.001; ****p < 0.0001

In the CFA-then-drug paradigm (Fig. 6a), drug treatment impacted pp38 (*F*_2,25_ = 8.013; *p* = 0.0020), IL-1β (*F*_2,24_ = 6.358; *p* = 0.0061), CGRP (*F*_2,25_ = 11.12; *p* = 0.0004), astrocyte activation (GFAP) (*F*_2,23_ = 4.285; *p* = 0.0262), and P2X7Rs (*F*_2,25_ = 3.713; *p* = 0.0401). Microglial (OX42) differences were not apparent at this time point (*F*_2,25_ = 1.029; *p* = 0.3719).

**Fig. 7:**
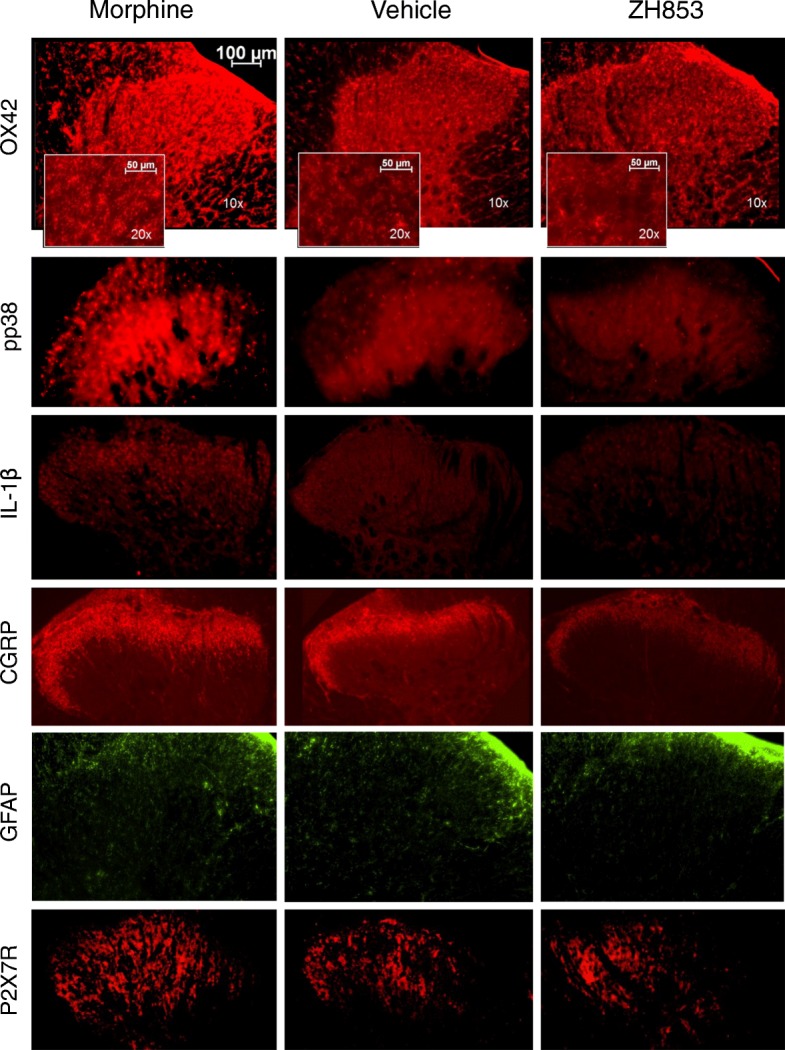
Representative images of IHC from the Drug-then-CFA paradigm. Each analysis was done on images acquired with the same exposure, gain, and aperture across treatment groups, but exposure and gain were adjusted for each antibody. All images were taken on a × 10 objective and microglia were imaged at × 20 for the inset of OX42. OX42 labels microglia, pp38 labels phosphorylated p38 Map Kinase, IL-1β labels interleukin-1beta, CGRP labels α-calcitonin gene-related peptide, Astro6 labels glial fibrillary acidic protein (GFAP), and P2X7R labels the P2X purinoceptor 7

In the drug-then-CFA paradigm (Fig. 6b), drug greatly impacted microglial activation (OX42) (*F*_2,19_ = 6.891; *p* < 0.0001), pp38 (*F*_2,21_ = 9.140; *p* = 0.0014), IL-1β (*F*_2,21_ = 10.86; *p* = 0.0006), CGRP (*F*_2,21_ = 10.35; *p* = 0.0007), astrocyte activation (GFAP) (*F*_2,21_ = 12.57; *p* = 0.0003), and P2X7Rs (*F*_2,20_ = 9.328; *p* = 0.0014). Representative images for this paradigm for all markers are included in Fig. 7.

The effect of CFA alone is represented by the vehicle-treated group. Except for OX42 in the CFA-then-drug model (Fig. 6a), morphine increased all inflammatory markers relative to ZH853. pp38 was reduced by ZH853 relative to vehicle; otherwise, ZH853 and vehicle groups were not significantly different. Morphine increased IL-1β and CGRP relative to vehicle. In the drug-then-CFA model, morphine increased all inflammatory markers relative to ZH853. ZH853 reduced IL-1β and GFAP versus vehicle, and morphine increased OX42, CGRP, and P2X7Rs versus vehicle.

**Fig. 8:**
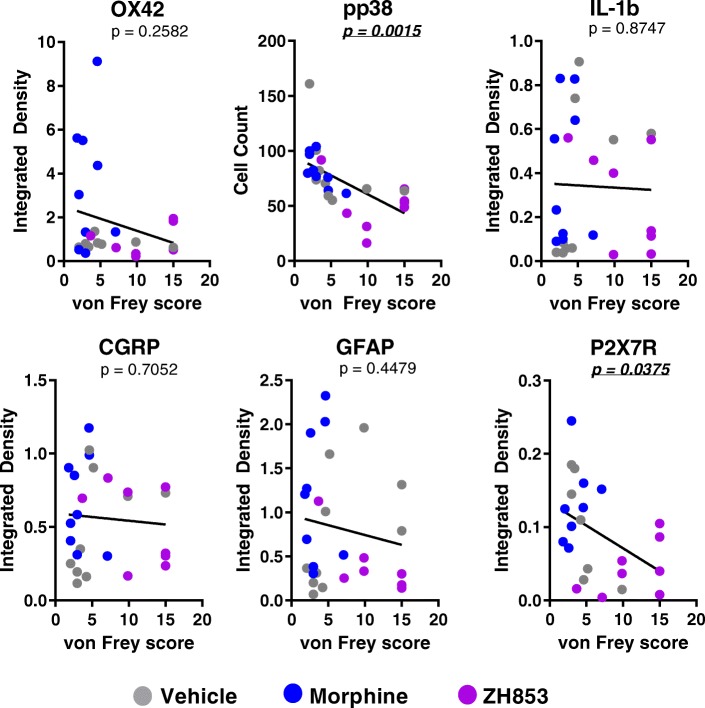
Regression analyses of IHC markers versus von Frey scores. Individual scores for both von Frey at the time point just prior to perfusion and integrated density (or cell count for pp38) from both paradigms of CFA and drug treatment were plotted for all drug groups. pp38 and P2X7R are consistently correlated with pain. *P* values are listed on each graph

A simple linear regression was calculated to determine whether a correlation existed between inflammatory markers and von Frey scores regardless of drug administered or paradigm (Fig. 8). A significant positive correlation with mechanical allodynia was found for pp38 (*F*_1,24_ = 12.87, *p* = 0.0015), with an *R*^2^ of .3490, and for P2X7R (*F*_1,21_ = 4.930, *p* = 0.0375), with an *R*^2^ of 0.1901. There were no significant correlations between allodynia and OX42, IL-1β, CGRP, or GFAP at these time points.

### Latent sensitization

While we expected that morphine-treated animals would experience greater LS compared to vehicle-treated animals, there appears to have been a “bottoming out” effect after naltrexone administration, causing both groups to appear equally affected. However, ZH853-treated animals consistently did not experience LS unmasking regardless of type of pain or order of drug treatment.

**Fig. 9:**
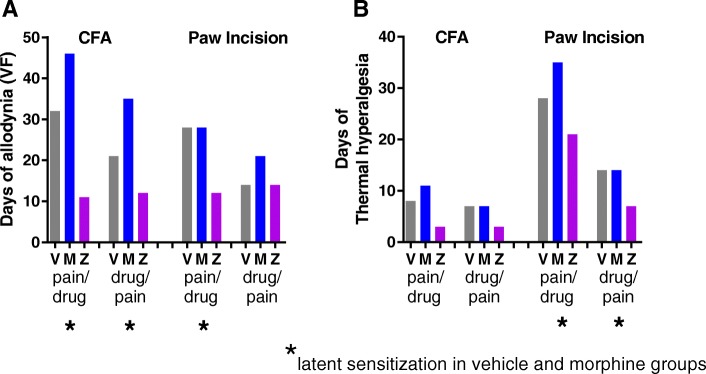
Total number of days in pain. **a** The number of days that rats experienced allodynia by pain type and drug/pain sequence. **b** The number of days that rats experienced thermal hyperalgesia by pain type and drug/pain sequence. Asterisks indicate which scenarios produced LS in the morphine and vehicle groups

Corder et al. [4] described the unmasking of LS following recovery from CFA in mice and showed that both mechanical allodynia (von Frey) and thermal hyperalgesia (Hargreaves) scores indicated a relapse to pain. We were surprised to find that in both paradigms of the CFA model, LS unmasking was apparent in von Frey testing (Figs. 2a and 3a) but not in the Hargreaves test, even with a 5 mg/kg naltrexone dose (Figs. 2b and 3b). After paw incision then drug treatment, allodynia was apparent in vehicle- and morphine-treated groups, but there was no relapse to allodynia in the reverse paradigm (Fig. 4b). However, relapse to thermal hyperalgesia was clearly present in both paradigms of paw incision (Fig. 5b). To see if there was any connection between the lengths of time in pain and whether or not LS developed, we plotted a summary of the total number of days in pain, charted according to pain type and sequence of drug and pain timing (Fig. 9). The total number of days in pain were calculated by two-way ANOVA as described in Tables 1, 2, 3, and 4. In addition, asterisks mark the instances where vehicle- or morphine-treated animals showed LS unmasking after naltrexone was administered. It is visually clear that LS occurred in instances where there was prolonged pain, but not if pain was under 21 days for allodynia (Fig. 9a) or under 11 days of thermal hyperalgesia (Fig. 9b). ZH853-treated animals consistently experienced less time in a hypersensitive state than other groups, which could be a major contributing factor to ZH853’s protective effect against LS.

## Discussion

The results of this study show that chronic use of ZH853 does not exacerbate the hyperalgesia and allodynia induced by inflammatory or post-operative pain while morphine does. Consistent with prior literature [1, 2, 16, 17], morphine exposure at moderately antinociceptive doses caused increased pain sensitivity. ZH853, which produced antinociception with less tolerance than morphine, caused no such increase in pain sensitivity across all pain tests. In fact, ZH853 diminished the amount of time in pain versus morphine in all tests and even versus vehicle treatment in several instances. This was an unexpected and unprecedented finding considering that opioids are known to increase and prolong many types of pain [1, 2, 16, 17]. Finally, ZH853 protected against the development or susceptibility to LS while morphine did not.

In addition to classical pain tests, we assessed operant behavior using the CatWalk and found that injury-induced deficits were transient and detectable at earlier time points than in the classical von Frey and Hargreaves tests. Significant impairment of gait by morphine, but not ZH853, was observed in several parameters during the drug-then-CFA paradigm. Gait analysis for pain monitoring is becoming increasingly developed for use in humans [34, 35] and, similarly to the animals in this study, pain-inducing inflammatory conditions of the human foot result in a loss of heel strike [35]. Antalgic (pain-avoidance) gait is defined by a reduced stance phase relative to swing phase, which is observed in humans with pain as well as animals [28, 35, 36] and observed in both paradigms of this study following CFA (Figs. 2c and 3c). In the pain-then-drug paradigm (Fig. 2c), drug dosing began as functional impairments were nearly back to baseline, which was likely the reason that no differences were apparent between drug groups. In the drug-then-pain paradigm (Fig. 3c), animals exposed to morphine showed greater gait impairments across several parameters up to the 7th day after CFA injection, indicating a decreased use of the left hind foot (guarding) versus vehicle- and ZH853-treated animals. The drug-then-CFA paradigm was the only test that captured a drug-induced difference in sensitivity within the first few days. For the drug-then-paw incision test, a less robust hypersensitivity, and therefore smaller dynamic range of responses, may have masked differences. For both the CFA and paw incision then drug tests, recovery had occurred by the end of dosing. The fact that functional impairments were observed from day 1 after CFA until day 3 or day 7 but not after that suggests that this test is not measuring mechanical allodynia as some groups have proposed [31]. The recovery timelines observed in this study mimicked those previously described in mice [18] and rats [37], showing short-term functional impairments (guarding, spontaneous pain) and long-term increases in allodynia and thermal hyperalgesia. In a comprehensive study by Djouhri et al. [38], spontaneous foot lifting (SFL) was used to measure spontaneous pain in comparison to von Frey and Hargreaves testing after CFA injections. In the first 2 days after injury, rats exhibited significant SFL which did not correlate with von Frey scores. However, they found that spontaneous C-fiber activity had greatly increased in these early time points. Although not directly compared in this study, it is not unreasonable to suggest that gait impairments are indicative of spontaneous pain, but many investigators have commented on the inability of painkillers to reverse these gait impairments acutely. Interestingly, some aspect of prior morphine, but not ZH853, managed to make these gait impairments worse, possibly through an increase in spontaneous activity at Aδ and/or C-fibers [39], which was not directly tested here. Finally, naltrexone was unable to provoke antalgic gait changes, which may differentiate this phase of pain from allodynia or thermal hyperalgesia that are susceptible to relapse (LS).

Rats treated with morphine generally showed signs of increased spinal inflammatory markers in immunohistochemical tests when compared to vehicle- and ZH853-treated animals. This supports findings from several other studies [1, 2, 11, 16, 17, 40]. While vehicle-treated animals usually had inflammatory expression somewhere between ZH853- and morphine-treated animals, ZH853-treated animals consistently showed the least amount of inflammation.

**Fig. 10:**
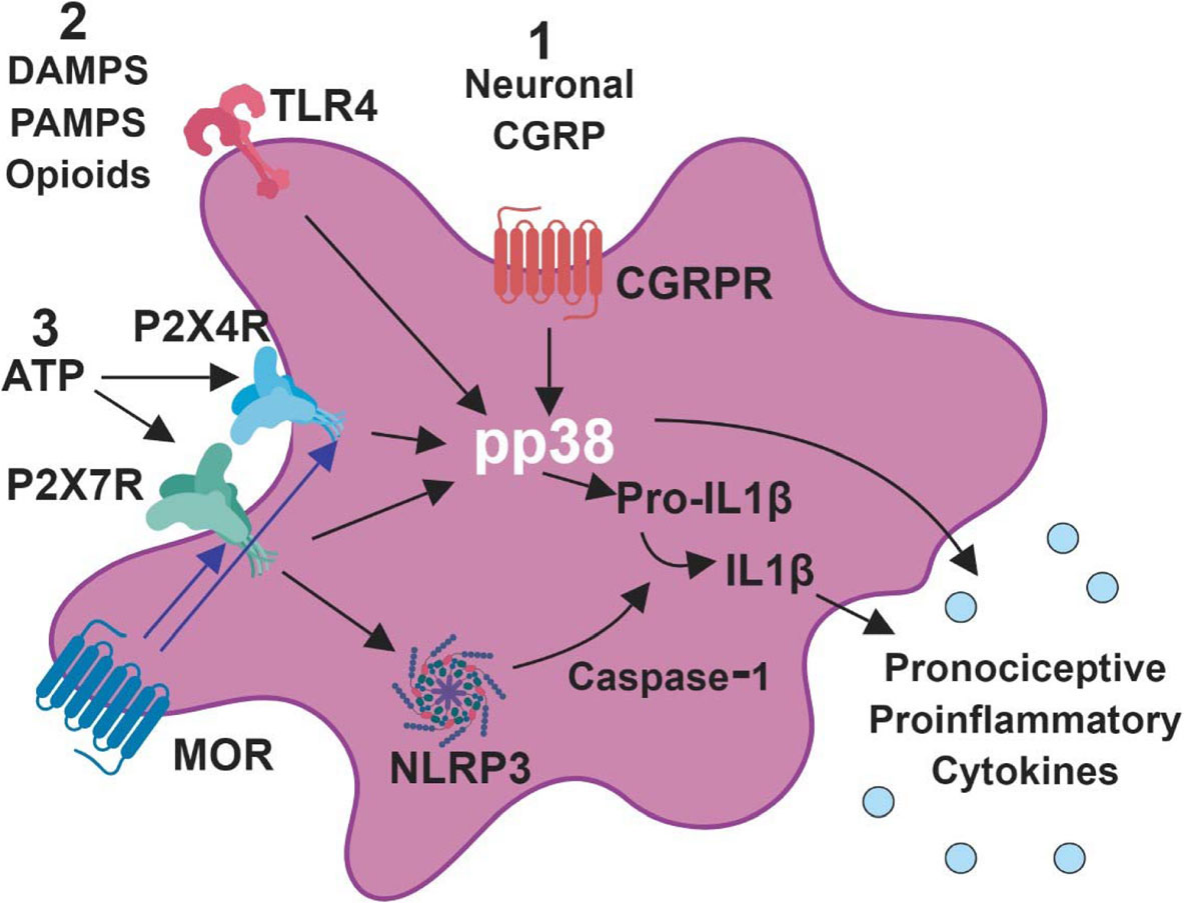
Schematic of postulated mechanisms of microglial activation by morphine but not by ZH853. Proposed mechanisms by which morphine activates glia or enhances trauma-induced activation include (1) induction of neuronal CGRP that activates microglial CGRP receptors [45], (2) binding at toll-like receptors (TLRs) alone or in concert with damage- or pathogen-associated molecular patterns (DAMPs, PAMPS) [46], and (3) activation of purinergic P2X receptors including by upregulation through MOR [47] (but see [48]). Each of these mechanisms causes phosphorylation of the mitogen-activated protein kinase (MAPK) p38 to increase transcription and translation of cytokines, including interleukin-1β (IL-1β [49], example shown), P2X7R activation also induces NOD-like receptor family, pyrin domain containing 3 (NLRP3) to become a complex (inflammasome) [50] that contains and releases active caspase-1 which cleaves pro-IL-1β into the active cytokine IL-1β [51]. IL-1β activates IL-1 receptors on astrocytes and neurons, further increasing inflammation. Ultimately, this proinflammatory pathway causes dorsal horn plasticity that leads to central sensitization and increased pain [52]

Previous reports from our lab and others have shown pro-inflammatory effects of morphine [11], and additive inflammatory effects of morphine in an animal in pain [1, 2, 16, 17]. Anti-inflammatory drugs like minocycline can block the development of tolerance, addiction, and dependence [41–44], but in humans it is not always possible or beneficial to treat with these drugs long-term. Several mechanisms by which morphine activates glia or enhances trauma-induced activation have been proposed (Fig. 10), including (1) induction of neuronal CGRP that activates microglial CGRP receptors [45], (2) binding at toll-like receptors (TLRs) alone or in concert with damage-associated molecular patterns (DAMPs) [46], and (3) activation of purinergic P2X receptors including by upregulation through MOR [47] (but see [48]). Each of these mechanisms causes phosphorylation of the mitogen-activated protein kinase (MAPK) p38, which activates nuclear factor kappa-light-chain-enhancer of activated B cells (NFκB) to increase transcription and translation of cytokines, including interleukin-1β (IL-1β [49], example shown), as well as IL-6, IL-18, and tumor necrosis factor-α (TNF-α). P2X7R activation also induces NOD-like receptor family, pyrin domain containing 3 (NLRP3) to become a complex (inflammasome) [50] that contains and releases active caspase-1 which cleaves pro-IL-1β into the active cytokine IL-1β [51]. IL-1β activates IL-1 receptors on astrocytes and neurons, further increasing inflammation. Ultimately, this proinflammatory pathway causes dorsal horn plasticity that leads to central sensitization and increased pain [52]. Pre- and/or postoperative inhibition of TLR4, phosphorylated p38 (pp38), NFκB, and IL-1β have been shown to reduce postoperative mechanical hyperalgesia [53–56].

While chronic morphine also activates all of these pathways in naïve rats, we recently showed the ZH853 activates none of them in a pain-naïve state [11]. In pain states, preemptive use of EM-1 and EM-2 reduces inflammatory (CFA) pain for days, even though the analgesic actions of the EMs only last ~ 30 m [57]. Through a number of pharmacological manipulations, Zhang et al. determined that p38 activation was significantly increased by pain but dramatically reduced by the EMs through a MOR-dependent mechanism [57]. Additionally, they found EM-induced reductions in IL-1β, TNF-α, C-C motif chemokine ligand 2 and 3 (CCL2/3), with an increase in IL-10 mRNA.

Others have shown that chronic morphine upregulates CGRP [45, 58], but this is the first time that long-term upregulation of CGRP following morphine and pain has been observed. In both CFA paradigms, vehicle- and ZH853-treated animals had similar CGRP expression, but weeks after the cessation of drug administration and CFA, morphine-treated animals had significantly higher levels of CGRP. Although not a direct component of the immune system, CGRP has been identified as a key player in the neuro-immune axis [59] with a range of functions at T cells, dendritic cells, mast cells, and macrophages that modulate pro- or anti-inflammatory factors. In particular, CGRPRs on microglia are likely a site of immune cell priming by pain in the spinal cord [45] which is then exacerbated by morphine or vice versa.

Astrocytes have largely been neglected in the long-term pain and opioid studies mentioned here, but one study found that astrocyte activation, not microglia, is best correlated with allodynia over time after induction of neuropathic pain [60]. After postoperative CFA plus morphine, neither astrocytes nor microglia appeared to be activated in staining, but other markers (pp38) indicated that they were activated [16]. In the current study, reduced immunofluorescence of GFAP and other markers indicates an anti-inflammatory effect of ZH853 versus the morphine-treated group (Fig. 6a). In the drug-then-CFA paradigm, ZH853 is anti-inflammatory versus the vehicle-treated group (GFAP, IL-1β) and, to a greater degree, versus the morphine-treated group (all markers) (Fig. 6b). However, astrocyte activation does not correlate with CFA-induced pain scores (Fig. 8).

Despite the seemingly clear relationship between increased inflammation and prolonged pain, pp38 and P2X7R are the only markers whose upregulation actually correlates directly with pain scores (Fig. 8). These findings underscore the utility of the pp38 marker in assessing pain pathways after complex manipulations; it is a unifying factor regardless of whether microglia are activated by CGRP, TLR4, or P2XRs. In addition, the results indicate that P2X7R may be of particular importance in CFA-induced pain. Given that ZH853 reduced all inflammatory factors versus morphine, it is reasonable to use the novel peptide in place of morphine to avoid production of additional pro-inflammatory, pronociceptive agitation in the CNS.

LS has been demonstrated in a number of pain types and animals [6, 7, 32, 61]. LS develops as a homeostatic mechanism to combat ongoing pain, allowing an organism to function effectively in the face of pain that has outlasted utility. Chen et al. discovered that top-down initiation of MOR_CA_ is blocked by spinal lidocaine administration [3], indicating that initiation comes from the brain in response to long-lasting pain. It appears that ZH853, either prior to or after pain induction, acts to quiet pain signaling in a manner distinct from morphine, alleviating the need for development of a compensatory mechanism (MOR_CA_). When the pain insult finally resolves, there is no underlying pathology in place that is susceptible to unmasking by naltrexone. Morphine, on the other hand, continues to exacerbate pro-inflammatory, pro-nociceptive signaling that ramps up the pain signal. This in turn ramps up the compensatory MOR_CA_, allowing LS to manifest. Additionally, opioids like morphine induce LS in the absence of pain in an *N*-methyl-d-aspartate receptor (NMDA)-dependent manner [5], and one group has found that N_2_O can prevent the development of opioid-induced LS by acting as an NMDA antagonist [62]. While some unpublished work in the lab has indicated that ZH853 does not bind significantly to NMDARs, no studies have been done to determine whether ZH853 acts as an NMDA allosteric modulator, but this is a possible explanation for the results described in this section.

As shown in Fig. 9, LS was produced in five of the eight paradigms tested. Those not producing LS were conditions in which the days in pain were generally shorter. This suggests that perhaps the severity and length of pain signaling has an influence on the development of LS and ZH853 might be protective against LS simply because ZH853-treated animals spent less time in pain overall. However, postoperative thermal hyperalgesia (Fig. 9b 3rd column) was the exception to this with ZH853-treated animals experiencing 21 days in pain with no sign of LS, indicating that factors other than time in pain contribute to both LS and its blockade by ZH853.

A drug that prevents the transition from acute to chronic relapsing pain would represent a true breakthrough in drug development for pain management. Not only have the mechanisms behind the shift from acute to chronic pain been elusive, but efforts to thwart this transition have been unsuccessful thus far. Two recognized markers of this transition are sustained glial activation [1, 2, 16, 43, 46, 63–68] and development of LS [4, 6, 8, 10, 62], which were both reduced by ZH853.

## Conclusion

Chronic pain patients and their doctors often face the dilemma of whether to risk serious side effects to achieve needed pain relief with opioids. Here, we show that the novel opioid, ZH853, shortens recovery time from inflammatory and postoperative pain while morphine prolongs recovery. Further, morphine does not affect latent sensitization while ZH853 blocks it. These results indicate two mechanisms of reduced transition from acute to chronic pain after treatment with ZH853. In addition, previous studies showed significant reduction, relative to morphine, of respiratory depression, abuse liability, tolerance, inflammation, and impairment of motor coordination [11] and equal or greater relief of pain in multiple chronic pain models [12]. Taken together, these results indicate that ZH853 could transform pain management.

## Abbreviations

ANOVA: Analysis of variance
Astro6: GFAP antibody
AUC: Area under the curve
CCI: Chronic constriction injury
CFA: Complete Freund’s adjuvant
CGRP: Calcitonin gene-related peptide
CNS: Central nervous system
DAMPs: Damage-associated molecular patterns
EM: Endomorphin
GFAP: Glial fibrillary acidic protein
i.t.: Intrathecal
IHC: Immunohistochemistry
IL: Interleukin
LH: Left hind
LS: Latent sensitization
MOR: Mu-opioid receptor
MOR_CA_: Mu-opioid receptor constitutive activity
NFκB: Nuclear factor kappa-light-chain-enhancer of activated B cells
NLRP3: Nucleotide-binding oligomerization domain, leucine rich repeat and pyrin domain containing 3 (inflammasome complex)
NMDA(R): *N*-methyl-d-aspartate (receptor)
NTX: Naltrexone
OX42: Anti-CD11b/c antibody (microglial marker)
P2X: Purinergic receptors- e.g., P2X4, P2X7
PBS: Phosphate-buffered saline
PEG: Polyethylene glycol
pp38: Phosphorylated-p38 MAP Kinase
RH: Right hind
SEM: Standard error of the mean
SFL: Spontaneous foot lifting
TLR: Toll-like receptor
